# Elite long jumpers with below the knee prostheses approach the board slower, but take-off more effectively than non-amputee athletes

**DOI:** 10.1038/s41598-017-16383-5

**Published:** 2017-11-22

**Authors:** Steffen Willwacher, Johannes Funken, Kai Heinrich, Ralf Müller, Hiroaki Hobara, Alena M. Grabowski, Gert-Peter Brüggemann, Wolfgang Potthast

**Affiliations:** 1 Institute of Biomechanics and Orthopaedics, German Sport University Cologne, Am Sportpark Müngersdorf 6, 50933 Cologne, Germany; 2 Institute of Functional Diagnostics, Im Mediapark 2, 50670 Cologne, Germany; 3 University of Applied Sciences Koblenz – RheinAhrCampus, Joseph-Rovan-Allee 2, 53424 Remagen, Germany; 4 ARCUS Clinics Pforzheim, Rastatter Str. 17-19, 75179 Pforzheim, Germany; 5 Human Informatics Research Institute, National Institute of Advanced Industrial Science and Technology, 2-3-26, Aomi, Koto-ku, Tokyo, 135-0064 Japan; 6 Integrative Physiology Department, University of Colorado Boulder, 354 UCB, Boulder, CO 80309-0354 USA; 7 Department of Veterans Affairs, Eastern Colorado Healthcare System, Denver, CO USA

## Abstract

The use of technological aids to improve sport performance (‘techno doping’) and inclusion of Paralympic athletes in Olympic events are matters of ongoing debate. Recently, a long jumper with a below the knee amputation (BKA) achieved jump distances similar to world-class athletes without amputations, using a carbon fibre running-specific prosthesis (RSP). We show that athletes with BKA utilize a different, more effective take-off technique in the long jump, which provided the best athlete with BKA a performance advantage of at least 0.13 m compared to non-amputee athletes. A maximum speed constraint imposed by the use of RSPs would indicate a performance disadvantage for the long jump. We found slower maximum sprinting speeds in athletes with BKA, but did not find a difference in the overall vertical force from both legs of athletes with BKA compared to non-amputees. Slower speeds might originate from intrinsically lower sprinting abilities of athletes with BKA or from more complex adaptions in sprinting mechanics due to the biomechanical and morphological differences induced by RSPs. Our results suggest that due to different movement strategies, athletes with and without BKA should likely compete in separate categories for the long jump.

## Introduction

Jumping for distance may be one of the most traditional competitive events in sports, with potentially the earliest use of performance enhancing technical aids (handheld weights); first performed during the Ancient Greek Olympic Games^1^. Following current competition rules, the long jump is performed after a preceding approach run of self-selected distance. A successful long jump requires the maximization of controllable run-up speed followed by an efficient redirection of the centre-of-mass (CoM) velocity during the take-off step^2^. Today, long jumping is also an essential part of the competition program for Paralympic athletes with amputations using running specific prostheses (RSPs) that are made from carbon fibre. RSPs are attached to a rigid socket that encompasses the residual limb and are thus in series with, or beneath, the residual limb. Unlike biological legs and feet, RSPs provide no sensory feedback and no control, cannot flex for ground clearance, and do not have the ability to change stiffness dynamically. RSPs allow for elastic energy storage and return, similar to tendons and ligaments of biological legs; but do not simulate the action of muscle fibres because RSPs cannot generate mechanical energy by conversion of metabolic energy.

Elastic mechanisms play an important role in animals specialized for jumping tasks^3–5^, because the power returned from elastic elements is nearly independent of speed^4,6^, as opposed to the power developed by muscle fascicles^7^. Therefore, it is reasonable to suppose that artificial limb designs featuring greater quantities of highly elastic components would perform better than their biological counterparts would during the take-off step in the long jump^8^. Indeed, the record distance of male athletes with below the knee amputation (BKA) using RSPs (8.40 m) has improved by 2.60 m (45%) since 1996; a resulting jump distance similar to those of current world-class non-amputee athletes, whose records have not changed during the same period. (Note: For the purposes of this article, it is assumed that athletes with BKA have a unilateral amputation and use a running-specific prosthesis below the site of amputation. It is also assumed that non-amputee athletes are not using any form of leg prosthesis or similar device.) The improved performances of athletes with BKA were observed after the introduction of carbon-fibre prostheses that are designed to mimic the spring-like behaviour of the biological lower extremities during running and sprinting^9^. Today, the best long jumpers with BKA take off from their affected leg using a prosthesis. All ten of the finalists in the 2016 Paralympic Games with BKA (class T44) took off from their affected leg using an RSP. The improved performance of long jumpers with BKA has led to speculation about a potential performance advantage compared to non-amputee athletes.

Athletes with BKA elicit lower anterior ground reaction forces and have longer contact times during the push-off from the starting blocks and the first step of the acceleration phase compared to non-amputees^10–12^. This indicates a performance disadvantage during maximum acceleration tasks compared to non-amputees; potentially due to the missing muscles and reduced capacity for positive power generation and other constraints imposed by the use of RSPs^10–13^. Still, no published data of athletes with BKA for subsequent steps of the acceleration phase exist at the moment. Furthermore, long jumpers do not accelerate maximally because the length of the run-up allows athletes to achieve maximum run-up speed over a longer period of time and distance. Thus, theoretical limits on force and power needed to accelerate maximally are likely to have little effect on long jump performance. Correspondingly, the limits on maximum constant sprinting speed appear to be more important for long jump performance^2^.

Previous studies suggest that maximum constant sprinting speed is primarily limited by the ability to apply high vertical forces to the ground during progressively shorter periods of ground contact with increasing speed^14,15^. Vertical impulse during the stance phase, the integral of force with respect to time, must be sufficiently high so that it yields aerial phases long enough for repositioning of the swing legs^14^. As running speed increases and consequently ground contact times decrease, higher average vertical support forces (ASFs) are required in order to create sufficient vertical impulse. Therefore, maximizing ASFs during short contact times is crucial for attaining fast maximum sprinting speed.

**Figure 1:**
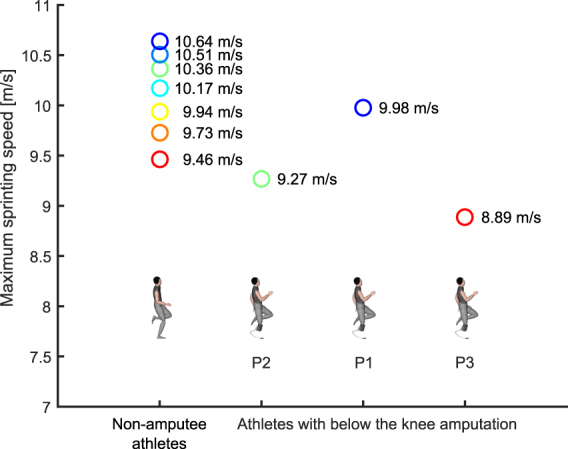
Maximum sprinting speeds observed in non-amputee athletes and athletes with below the knee amputation. Each data point represents the fastest speed obtained for each individual athlete. We use the same colors for individual athletes with BKA and non-amputee athletes throughout the entire article.

**Figure 2:**
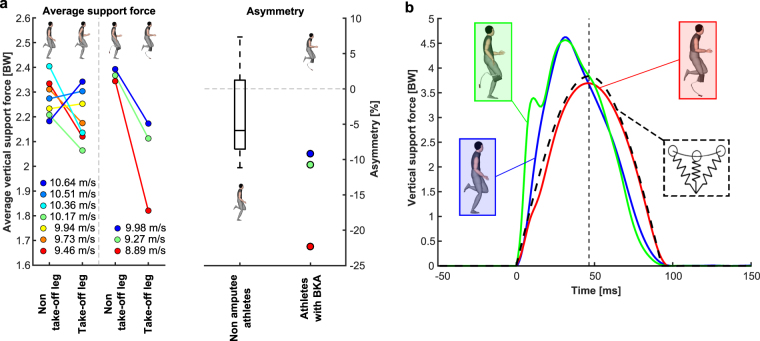
Maximum sprinting speed mechanics. (**a**) Stance average vertical support force (left) and average support force asymmetry (right) for take-off and non-take-off legs of all subjects during maximum sprinting speed trials in units of bodyweight (BW). Values are provided as the average over all trials for the respective leg. Negative asymmetry values indicate greater force applied by the non-take-off leg. (**b**) Representative vertical ground reaction force patterns of the fastest athlete with a below the knee amputation (BKA) and the fastest non-amputee athlete, compared to the pattern of an ideal spring-mass model calculated using the equations provided by Clark *et al*.^19^. The fastest athletes were also the athletes who achieved the longest distances during the long jump trials. For the spring-mass model, step, contact and aerial times from measurements of the same trial of the affected leg of the fastest athlete with BKA were used.

Athletes with BKA using RSPs have asymmetrical biomechanics between their affected and unaffected legs during constant speed running. For example, athletes with BKA exhibit 9% lower ASFs in their affected compared to unaffected leg across a wide range of speeds from 3 m/s up to top speed, and exhibit 18% lower leg stiffness in their affected compared to unaffected leg at 10 m/s^16,17^. Nonetheless, between-leg asymmetries in force application of 4.1% on average have also been reported during constant maximum speed treadmill sprinting of non-amputee sprinters^18^. The way that between-leg asymmetry affects overall ASF application demands on sprinters measured during several consecutive steps at maximum constant speed is currently unknown. Qualitative observation of representative waveform data provided e.g. in the work of Rabita *et al*.^13^ (page 5, Fig. 1) or Clark and Weyand^19^ (page 608, Fig. 2b), suggest that the ASF requirement is satisfied by considerable amounts of between-leg and step to step asymmetries. It is conceivable that lower ASF application in one leg can be compensated for by higher ASF application in the other leg. Still, the capacities for contralateral leg force application compensation at maximum sprinting speed are limited by biological constraints. Vertical forces averaged over both legs during consecutive steps would therefore be reduced if the use of an RSP during maximum speed sprinting induces an above threshold force impairment on the affected leg, which cannot be compensated for by the unaffected leg. This would imply a maximum speed limitation, based on the assumption that no additional compensations (i.e. higher step frequency or a longer distance travelled by the CoM during the contact phase) were present^14^.

We addressed the components that affect long jump performance by comparing the biomechanics of the world’s three best long jumpers with BKA from 2015 (personal record [PR], 7.43 ± 0.99 m), including the 2016 Paralympic champion (class T44), to a group of non-amputee long jumpers at a similar average performance level (n = 7; PR, 7.65 ± 0.65 m), including the 2016 Olympic champion, during maximum constant speed sprinting and maximum-distance long jumping. Our analyses were focused on factors previously associated with performance in sprinting and jumping, including ground reaction force production and the efficiency of CoM energy conversion during the take-off step. We hypothesized that athletes with BKA would have slower speeds during the approach run compared to non-amputees, due to force constraints imposed by their prostheses during maximum constant speed sprinting. In addition, we hypothesized that athletes with BKA would have more efficient energy conversion during the take-off step of the long jump compared to non-amputees, due to the elastic energy storage and return capabilities of their carbon fibre prostheses. Furthermore, we aimed to identify the motor solutions used by each group of jumpers by comparing the mechanical energy, both of the CoM and lower extremity joints.

## Results

### Maximum sprinting

During the sprinting trials, the best athlete with BKA had a 0.66 m/s (6.2%) slower maximum running speed compared to the fastest non-amputee athlete (Fig. 1). These athletes had very similar long jumping performance (7.96 m vs. 7.92 m, respectively). On average, athletes with BKA achieved a jump distance of 7.26 m ± 0.77 m, while athletes without BKA achieved 7.27 m ± 0.45 m. Athletes with BKA had 7.6% slower maximal sprinting speeds of 8.89–9.98 m/s (mean: 9.38 m/s), compared to non-amputee athletes, who achieved top sprinting speeds of 9.46–10.64 m/s (mean: 10.15 m/s) (p = 0.11, Fig. 1). We measured all maximum sprinting speeds using a laser gun.

Athletes with BKA applied 9.0% lower (p = 0.03) stance average vertical support forces (ASFs) to the ground with their affected leg compared to the average of left and right legs of non-amputees, while their unaffected legs applied 5.7% higher (p = 0.02) ASFs compared to the average of both legs of non-amputees (Fig. 2a, Table 1).

**Table 1:** Discrete parameters for the analysis of maximum speed sprinting.

	Long jumpers without amputations							Long jumpers with BKA					
	Mean	SD	Min	Max	P1 (7.96 m)			P2 (7.38 m)			P3 (6.43 m)		
					UL	AL	Mean	UL	AL	Mean	UL	AL	Mean
Maximum speed (m/s)	10.15	(0.42)	9.46	10.64	—	—	9.98	—	—	9.27	—	—	8.89
Stance averaged vertical GRF (BW)	2.24	(0.05)	2.14	2.29	2.39	2.17	2.28	2.37	2.11	2.24	2.34	1.82	2.08
Vertical impulse (BWs)	0.22	(0.01)	0.21	0.24	0.23	0.21	0.22	0.29	0.22	0.25	0.25	0.22	0.23
Step frequency (Hz)	4.45	(0.24)	4.10	4.72	—	—	4.34	—	—	3.85	—	—	—
Contact time (ms)	99	(7)	93	112	98	98	98	124	102	113	105	121	113
Contact length (m)	0.89	(0.07)	0.81	1.03	0.86	0.80	0.83	0.83	0.81	0.82	—	—	—
Swing time (s)	349	(18)	330	374	358	363	360	391	396	393	—	—	—
Negative work hip joint (J/kg)	0.87	(0.12)	0.73	1.02	1.32	0.25	0.79	1.03	0.10	0.57	—	—	—
Positive work hip joint (J/kg)	0.85	(0.24)	0.69	1.32	2.19	1.20	1.70	1.47	1.30	1.39	—	—	—
Negative work knee joint (J/kg)	0.56	(0.17)	0.35	0.87	1.24	0.19	0.72	1.00	0.14	0.57	—	—	—
Positive work knee joint (J/kg)	0.25	(0.11)	0.13	0.44	0.16	0.10	0.13	0.20	0.05	0.13	—	—	—
Negative work below knee joints (J/kg)	2.17	(0.23)	1.94	2.54	1.94	1.54	1.74	2.42	1.60	2.01	—	—	—
Positive work below knee joints (J/kg)	1.79	(0.16)	1.56	1.98	1.55	1.48	1.52	1.65	1.41	1.53	—	—	—

Stride frequency in athletes with BKA was calculated from complete stride cycles (including steps with the affected and unaffected legs). Stride frequency was then multiplied by two in order to calculate average step frequency. Kinematic data during sprinting could not be obtained from one non-amputee athlete and from one athlete with BKA. UL - unaffected leg of athletes with BKA. AL - affected leg of athletes with BKA. Mean - average value of affected and unaffcted legs of athletes with BKA. For non-amputee athletes averages of the left and right legs are presented. P1-P3: Long jumpers with BKA; their best jump distance achieved is provided in parentheses.

Directional asymmetries in ASF application between take-off and non-take-off legs were observed both in athletes with and without BKA (Fig. 2). The fastest athlete with BKA was 13% more symmetric than the slowest athlete with BKA (−9.2% vs. −22.2% asymmetry, respectively, Fig. 2a). Take-off leg vs. non-take-off leg (directional) asymmetry was calculated using the following formula:

1 $$Asymmetry\,( \% )=(\frac{{F}_{Avg\_Take\_off\_leg}-\,{F}_{Avg\_Non\_Take\_off\_leg}}{{F}_{Avg\_Non\_Take\_off\_leg}})\cdot 100$$

Here F_Avg_Take_off_leg_ and F_Avg_Non_Take_off_leg_ refer to the ASF created during the stance phase by the take-off and non-take-off leg, respectively. Negative values indicate a greater ASF application by the non-take-off leg while positive values indicate a greater ASF application by the take-off leg. Directional asymmetry between take-off and non-take-off legs tended to be higher in athletes with BKA compared to non-amputees (Fig. 2a).

When considering absolute values of between-leg asymmetry (fluctuating asymmetry, ignoring which leg was take-off and non-take-off leg), athletes with BKA displayed greater asymmetry than non-amputee athletes (14.1 ± 7.2% vs. 6.1 ± 3.8%, respectively), even though the level of significance was not reached (p = 0.12). Lower vertical force application in athletes with BKA appeared to be qualitatively closer to the behaviour of a spring-mass model, as indicated by the half- sinusoidal shape of the vertical ground-reaction force (GRF) curve (Fig. 2b). Recently, Clark *et al*. found that running speeds in elite sprinters are maximized by a vertical ground reaction force pattern that differs from the behaviour of classical spring-mass models^19^; this phenomenon was qualitatively observed during maximum speed sprinting for all of our non-amputee subjects, but not for the affected legs of athletes with BKA (Figs 2b; S1).

**Figure 3:**
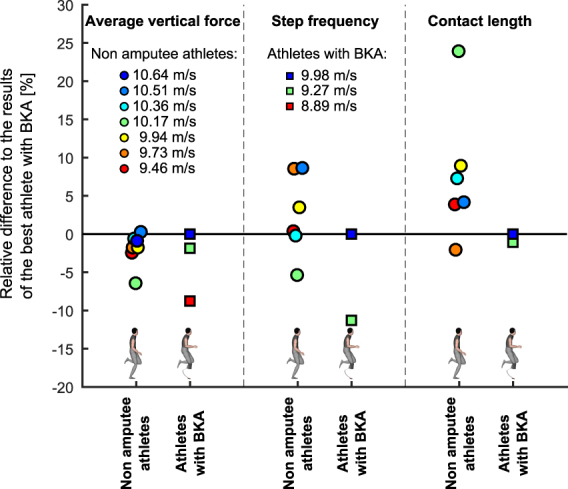
Individual results for average vertical force application from both legs, step frequency and contact length. Constant running speed equals the product of these three variables; thereby they indicate three potential mechanical approaches by which sprinters might achieve faster maximum speeds^14^. The graph shows the relative individual differences (in percent) compared to the results of the best athlete with below the knee amputation (BKA).

Nonetheless, when averaged across unaffected and affected legs, ASF in athletes with BKA was not different from non-amputee athletes (2.15 ± 0.13 BW vs. 2.24 ± 0.05 BW, respectively; p = 0.41; Table 1). The best athlete with BKA elicited 0.9% higher average ASF from both legs compared to the fastest non-amputee athlete and 2.0% higher between leg averaged ASF compared to the mean of all non-amputee athletes (Fig. 3, Table 1). Therefore, when averaging ASF from both legs during consecutive steps, there was not a general force reduction in athletes with unilateral BKA compared to non-amputees.

An explanation for the slower maximum speed observed in the best athlete with BKA despite similar average ASF application over consecutive steps might be found in the relatively low values of step frequency and contact length compared to non-amputee athletes with similar sprinting speed (Fig. 3, Table 1).

### Take-off step

The slower maximum sprinting speeds of athletes with BKA were also reflected in slower horizontal CoM velocities immediately before the take-off step (Table 2; Fig. S2). However, the best athlete with BKA and the best non-amputee athlete achieved very similar maximum jumping distances (7.96 m vs. 7.92 m). Jumping distance was calculated from the intersection of the parabolic flight curve of the CoM and the horizontal landing surface for all athletes, thereby ignoring any effects of the landing technique of the athletes (see methods section for details).

**Table 2:** Discrete parameters for the analysis of the long jump take-off step.

	Long jumpers without amputations				Long jumpers with BKA		
	Mean	SD	Min	Max	P1	P2	P3
					AL	AL	AL
Theoretical distance (m)	7.27	(0.45)	6.51	7.92	7.96	7.38	6.43
Horizontal velocity at touchdown (m/s)	9.39	(0.36)	8.86	9.86	9.32	8.61	8.22
Ratio touchdown/maximum velocity (%)	92.85	(2.31)	89.80	95.65	93.37	92.87	92.43
Vertical velocity at touchdown (m/s)	−0.37	(0.11)	−0.54	−0.23	−0.68	−0.52	−0.36
Contact time (ms)	125	(10)	108	141	118	127	147
Horizontal velocity loss (m/s)	−1.09	(0.23)	−1.49	−0.84	−0.64	−0.59	−0.57
Take-off angle (°)	17.98	(1.98)	16.41	20.91	18.25	20.10	16.94
CoM take-off height (m)	1.18	(0.06)	1.08	1.26	1.18	1.24	1.18
Horizontal CoM take-off position (m)	0.29	(0.06)	0.19	0.38	0.30	0.26	0.39
Vertical velocity at toe-off (m/s)	3.01	(0.26)	2.76	3.47	3.00	3.08	2.55
Vertical impulse (BWs)	0.41	(0.08)	0.30	0.52	0.48	0.49	0.43
Peak vertical force (BW)	8.05	(2.40)	5.26	12.21	6.35	6.03	4.44
Stance averaged vertical GRF (BW)	3.33	(0.60)	2.49	4.21	4.07	3.83	2.93
Net horizontal impulse (BWs)	−0.11	(0.03)	−0.17	−0.09	−0.07	−0.07	−0.05
Stance averaged resultant GRF (BW)	4.27	(0.81)	3.46	5.24	4.76	4.83	4.27
Ratio vertical/horizontal impulse	3.82	(0.54)	3.13	4.51	6.74	7.33	8.04
Total CoM energy at touchdown (J/kg)	53.71	(3.59)	48.73	57.78	53.72	47.72	43.64
Negative CoM work (J/kg)	−7.06	(1.13)	−9.14	−5.99	−5.33	−4.89	−2.96
Positive CoM work (J/kg)	3.99	(1.17)	2.21	5.50	5.33	6.18	3.38
Ratio positve/negative CoM work (%)	57	(16)	35	77	100	126	114
Negative work hip joint (J/kg)	2.66	(0.50)	2.07	3.56	0.16	0.17	0.35
Positive work hip joint (J/kg)	2.28	(0.76)	1.51	3.78	1.17	1.23	0.85
Negative work knee joint (J/kg)	2.56	(1.23)	1.62	5.21	0.91	0.64	0.74
Positive work knee joint (J/kg)	1.45	(0.50)	0.60	2.12	0.26	0.28	0.31
Negative work below knee joints (J/kg)	2.55	(0.81)	1.70	4.06	5.69	4.72	2.65
Positive work below knee joints (J/kg)	1.96	(0.28)	1.51	2.34	4.41	3.79	2.48

The result of the best jump (based on distance achieved) of each athlete was taken into consideration for the analysis. Center of mass is abbreviated with CoM throughout the table. AL - affected legs of athletes with BKA. P1-P3: Long jumpers with BKA.

**Figure 4:**
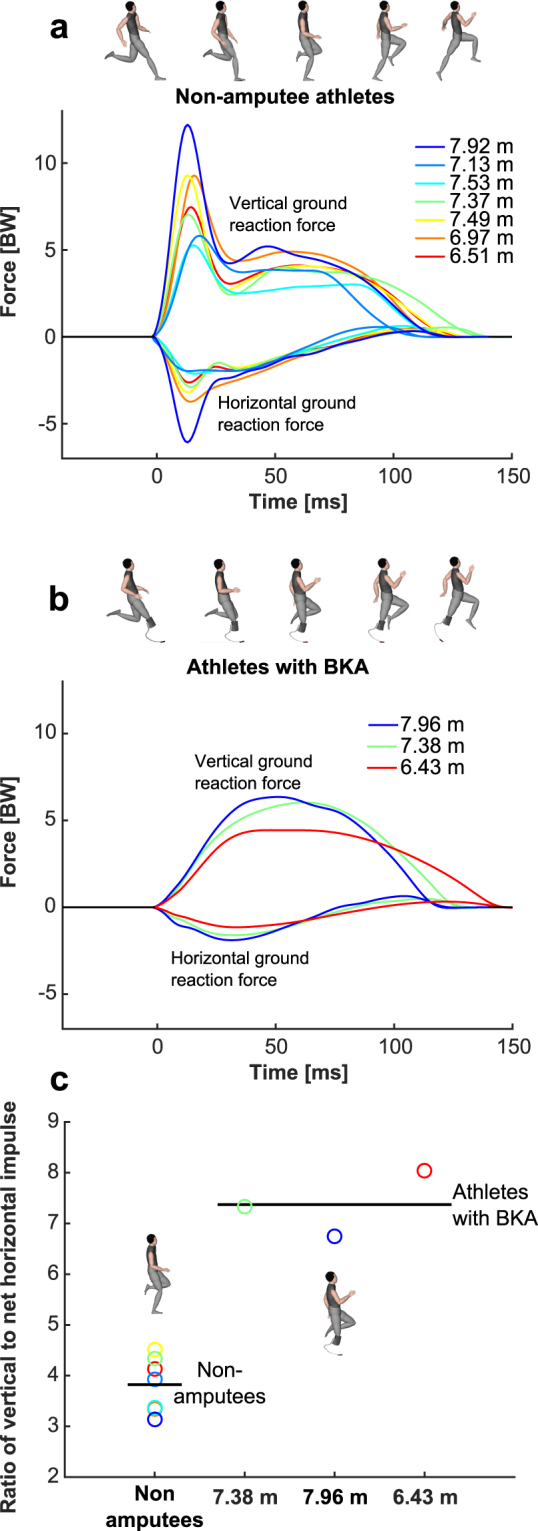
Take-off step mechanics. Vertical and horizontal ground-reaction forces (GRFs) for non-amputee athletes (**a**) and athletes with below the knee amputation (BKA) (**b**) in units of bodyweight (BW) for the take-off step of the long jump. Athletes with BKA lacked the initial vertical and horizontal (braking) peaks in their GRF curves. Note the simultaneous presence of the vertical and braking force peaks in non-amputee athletes. In athletes with BKA, GRF dynamics are qualitatively similar to the dynamics of an ideal spring-mass model^20,21^. Athletes with BKA generate a similarly large vertical impulse while generating a reduced net horizontal braking impulse compared to non-amputees, which results in higher ratios of vertical to net horizontal impulse (**c**), and a more effective take-off mechanism.

During the take-off step, all athletes with BKA lacked the initial impact-force peak in both the vertical and horizontal (braking) directions, so that the curves representing these forces closely resembled those produced by an ideal spring-mass-model^20,21^ (Fig. 4a,b). An effective take-off mechanism is characterized by the ability to create a large vertical impulse that launches the athlete into a parabolic flight curve, while at the same time avoiding a large horizontal braking impulse and the corresponding loss of CoM velocity and energy^2,8^ (Fig. 4). Athletes with BKA showed similar values for vertical impulses (p = 0.27, Table 1), but had lower net horizontal braking impulses (p = 0.02) and a corresponding lower horizontal velocity loss compared to non-amputees. This resulted in 93% higher values for the ratio of vertical to net horizontal braking impulse, indicating a more effective take-off mechanism for athletes with BKA compared to non-amputees (Table 2; Figs 4; S2). During the take-off step, athletes with BKA created similar ASFs compared to non-amputee athletes (p = 0.83, Table 2). The best athlete with BKA generated 3.3% lower ASF during the take-off step compared to the best non-amputee athlete (Table 2).

**Figure 5:**
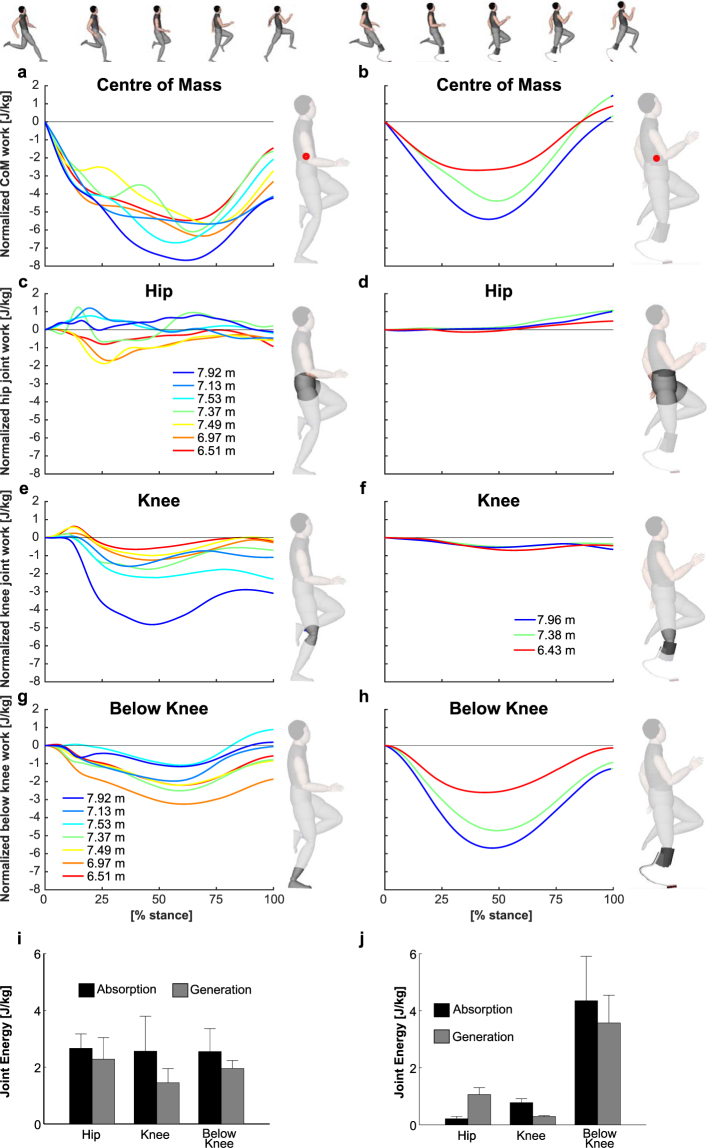
Stance-phase normalized work performed on the center of mass in non-amputee athletes (**a**) and athletes with below the knee amputation (BKA) (**b**). Joint work performed at the hip, knee and below the knee (ankle and metatarsal phalangeal joint) in non-amputees (**c**,**e**,**g**) and athletes with BKA (**d**,**f**,**h**). Below-knee work in athletes with BKA is the work performed by the prosthesis. Note the similarity between the centre of mass and below-the-knee work in athletes with BKA and the relatively low amounts of work performed at the hip and knee in athletes with BKA. Energy absorption and generation (mean + standard deviation) within the joints are summarized in the bottom part of the figure for non-amputees (**i**) and athletes with BKA (**j**).

The change in direction of the CoM trajectory during the take-off step involves conversion of some of the CoM energy into mechanically usable (i.e. elastic strain energy) and unusable forms of energy such as heat and sound^8,22^. During the first half of the take-off phase, non-amputee athletes reduced their CoM energy, and this energy was not fully regenerated during the second half of the take-off step (Fig. 5a).

The CoM energy generation of jumpers with BKA was 13.5 ± 13.1% greater than the energy absorbed during the first part of the contact phase (Fig. 5b; Table 2). Whereas, the CoM energy generation of non-amputee athletes was only 56.7 ± 15.9% of the energy absorbed during the first part of the contact phase. This corresponds to a net CoM energy gain of 0.57 ± 0.65 J/kg for athletes with BKA and a net loss of 3.07 ± 1.27 J/kg for the non-amputee athletes. CoM energy losses during the take-off phase were strongly correlated with reductions in horizontal kinetic energy (Pearson correlation, r = 0.96, p < 0.01), while vertical kinetic energy (r = −0.50, p = 0.14) and potential energy (r = −0.62, p = 0.06) were higher at the end of the take-off step than at the beginning (Fig. S3).

To better understand the sources of these differences in CoM energy absorption and generation, we analysed the work patterns of the major joints of the lower extremities^23,24^. Athletes with BKA utilized a distinctively different motor control strategy, which relied on storing and returning a larger amount of mechanical energy within the carbon-fibre prosthesis (Table 3; Figs 5h,j; S4). Comparatively small absolute portions of energy were absorbed at the knee and generated at the hip in athletes with BKA compared to non-amputees (Tables 2 and 3; Figs 5d,f,j; S4).

**Table 3:** Negative (neg.) and positive (pos.) work at the individual joints as a % of CoM negative work and positive work, respectively.

	Relative work (% CoM work)	Long jumpers without amputations				Long jumpers with BKA		
		Mean	SD	Min	Max	P1 (7.96 m)	P2 (7.38 m)	P3 (6.43 m)
						AL	AL	AL
Neg. work	Hip	38.44	(9.20)	28.50	51.46	3.00	3.48	10.47
	Knee	35.04	(10.26)	26.42	56.99	17.09	13.09	26.35
	Below knee	37.28	(13.31)	19.91	58.72	106.85	96.51	89.18
Pos. work	Hip	59.43	(16.83)	32.59	79.28	21.96	19.92	23.36
	Knee	39.42	(16.33)	11.83	56.02	4.88	4.53	9.76
	Below knee	53.26	(20.87)	36.42	98.73	82.77	61.38	74.23

AL - affected legs of athletes with BKA. P1-P3: Long jumpers with BKA.

The net CoM energy increase during the take-off step of athletes with BKA could therefore be explained by the additional positive muscular work at the hip, which added to the energy returned from the prosthesis. Energy absorption and generation were more evenly distributed between joints in non-amputee jumpers compared to athletes with BKA during the take-off step (Tables 2 and 3; Figs 5c,e,g,i; S4).

## Discussion

The purpose of the present study was to identify the key biomechanical differences in maximum effort long jumps between athletes with BKA and non-amputee athletes. This was necessary as the existing research^25–27^ does not include performances at the level recently observed for the best athletes with BKA and/or only includes kinematic analyses, which are not able to distinguish kinetic differences at the joint level. Nonetheless, these analyses are needed to justify any conclusions regarding motor control solution similarity between athletes with BKA and non-amputees.

A strength of the present study is that it potentially includes the best athlete with BKA and one of the best non-amputee athletes at the time of data collection. This can be observed from their personal records (8.40 m vs. 8.52 m, respectively) and from their long jump results during the experiment (7.96 m vs. 7.92 m, respectively). Therefore, a direct comparison of the maximum performances currently observed in humans with and without BKA was possible, although no inferential statistics could be applied when comparing the results of these two athletes directly. Furthermore, the biomechanical differences in motor solution strategies could be compared in jumps with very similar performance outcomes (i.e. jump distance).

In the present study, athletes with BKA demonstrated slower maximum speeds during the sprinting trials (Fig. 1), as well as slower horizontal speeds immediately before the take-off step compared to non-amputee athletes, while the ratio between the two speeds was not different between groups (Table 2). A constraint on maximum running speed induced by the use of RSPs would indicate a performance disadvantage for the long jump. Maximum constant running speed is among other factors related to the ability to apply high vertical GRFs in short periods of time, which results in high ASF application on the ground^14^. This is necessary to facilitate sufficient aerial times in order to reposition the swing leg for the next step. Furthermore, when accelerating up to maximum speed, high forward directed ground reaction force and power are needed to increase the horizontal velocity of the athlete. Better sprinters keep the GRF vector oriented forward for a longer period of time^28^, thereby reaching higher maximum constant speed.

We found that athletes with BKA had lower ASFs elicited by their affected compared to unaffected leg during maximum constant speed sprinting, which led to higher ASF asymmetry compared to their non-amputee counterparts. The 9% reduction in ASF during sprinting for the affected compared to unaffected leg of the best athlete with BKA in this study matches nicely with the average ASF reductions in the affected compared to unaffected leg reported previously for elite athletes with BKA^16^. These lower ASFs may be related to differences in the limb posture required to run using a prosthesis and a reduced ability to create high leg stiffness when using a prosthesis^17^. Theoretically, a number of additional reasons for the identified ASF asymmetry exist, which could include the minimization of overall biomechanical asymmetries, improvement of balance and limb-socket comfort, and/or the differences in the decelerated effective mass below the knee during impact^29^. These influences on ASF symmetry need to be investigated in future studies in greater detail.

**Figure 6:**
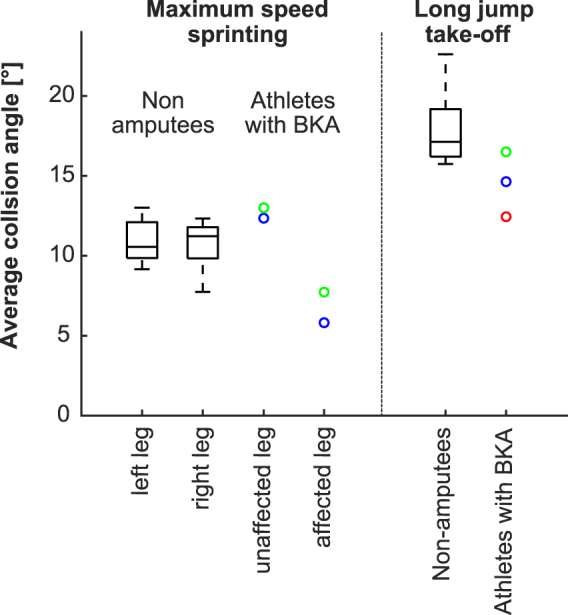
Average collision angles determined through the energy absorption phase of ground contact during maximum-speed sprinting and the long jump take-off step. As motion capture data were not available for the sprinting trials of the third best athlete with below the knee amputation (BKA), sprinting data from only two subjects with BKA are shown. In general, the greatest collision angle occurred during the take-off step for the long jump, in both sets of athletes. The best long jump performance coincided with the greatest collision angle.

Inferring a general reduction of ASF application due to the use of RSPs is not possible, as the average ASFs during the take-off step (3.61 ± 0.60 BW) clearly exceeded the average ASFs during sprinting (2.16 ± 0.22 BW). This indicates that athletes with BKA are capable of creating higher ASFs in tasks different from maximum speed sprinting. On the other hand, during sprinting and jumping, the constraining conditions under which forces are generated are dissimilar. Maximum, constant speed sprinting requires a sufficient vertical impulse, which allows an aerial time long enough to reposition the leg for the next step. The major demands of maximum speed sprinting are to generate high vertical forces during progressively shorter contact times and to generate slightly higher propulsive than braking impulse in order to keep a constant running speed while overcoming air resistance. The take-off step requires a high vertical impulse while keeping the net horizontal impulse as low as possible. Accordingly, the conditions for force application are different in the two conditions, which is reflected in the longer contact times and greater collision angles observed during the take-off step for all athletes (Table 2, Fig. 6). Furthermore, differences with respect to the external GRF lever arms at the lower extremity joints between sprinting and the take-off step are conceivable^15^. Consequently, higher stance average forces were measured during the take-off step in all athletes taking part in the study, including non-amputee athletes (Table 2, Figs 2 and 3). Future studies should explore the conditions that affect the application of force on the ground in different situations.

Fluctuating morphologic asymmetry is negatively correlated with sprint speed and racing ability in cursorial animals like lizards^30^, racehorses^31^ and humans^32^. Between-leg asymmetry resulting from impaired ASFs on the affected side might be related to functional and morphological differences introduced by the RSP and has been attributed to be a limiting factor in achieving top speeds compared with non-amputee sprinters^16^. The results of the present study suggest that athletes with BKA can compensate for reduced ASF on their affected side by generating higher ASF on their unaffected side during maximum speed sprinting. This was clearly the case in the best athlete with BKA, whose between leg averaged ASF was higher compared to the best non-amputee long jumper and the mean value of non-amputees during maximum speed sprinting (Table 1). Furthermore, the between-leg averaged ASFs of all athletes with BKA fell nicely within the relationship between running speed and ASF application provided in the literature^14^. We also observed asymmetries of 6% on average in our non-amputee athletes, which was slightly higher than the 4.1% ASF asymmetry reported in a recent study of non-amputee treadmill sprinting^18^. Therefore, it is likely that non-amputee athletes use similar compensation strategies to meet the ASF demands at their top running speeds. From an average ASF application point of view, the use of RSPs seems to induce no general performance limitation during maximum speed sprinting. As mentioned above, this is in disagreement with the interpretation of a previous study^16^. Their experimental design did not have a non-amputee control group, thus they did not compare ASF from both legs of athletes with BKA to those of non-amputees. Our results show clear differences in ASF asymmetry between athletes with BKA of different performance levels. Higher between-leg ASF asymmetries were found in slower compared to faster athletes with BKA. If the use of an RSP in an individual athlete results in between leg ASF asymmetry above a certain threshold which cannot be compensated for by the unaffected leg, then this might result in a slower run-up speed. Therefore, improving biological force application capacities and utilizing a running technique that minimizes between leg asymmetries and the necessity for ASF compensation appear to be two key strategies to improve maximum sprinting speed in long jumpers with BKA. Nonetheless, the specific threshold of between leg asymmetry, above which a compensation by higher contralateral ASF application becomes impossible, is currently unknown and should be determined in future studies.

During the take-off step, we found that athletes with BKA utilized a technique that allowed them to store and return more energy in their RSPs compared to the legs of non-amputees. Beneficial conditions for power generation by means of energy storage and return within the tendons and ligaments of biological limbs play an important role in jumping^3–6,8,33^. In this context, beneficial conditions for power generation refer to a higher power output from elastic structures as compared to power developed by muscle fascicles alone, because power developed from elastic structures is not constrained by the muscle fascicle’s power-velocity relationship. The human Achilles tendon can store 0.51 J/kg during running at 3.9 m/s^34,35^, while in full-effort sprinting, 0.70 J/kg of positive work is performed by reutilizing the strain energy in the Achilles tendon^36^. In addition, the human foot is capable of storing about 17 J of strain energy (around 0.21 J/kg for someone weighing 80 kg) when forces similar to running at 4.5 m/s are applied^35^. Other tissues like the long tendons of extrinsic foot muscles and the ankle-joint ligaments are capable of storing additional energy, though the quantities of elastic energy are lower than for the Achilles tendon and the foot. No measured energy storage values have been reported for the maximal dynamic motions of elite long jumpers, so direct comparison of passive elastic strain energy storage capacities between biological and prosthetic legs may not be applicable. The theoretical upper limit to energy return from elastic structures within the leg can be calculated by assuming that all positive work performed at and below the knee is the result of passive energy return^37^. The best athlete with BKA generated 37% (1.27 J/kg) and 12% (0.50 J/kg) more positive work at and below the knee than the non-amputee average and the best non-amputee long jumper (PR: 8.52 m), respectively (Table 1), during the take-off step. This indicates that the amount of elastic energy storage and return from prosthetic limbs may not be achievable for non-amputee athletes taking off from their biological limbs and therefore, use of a prosthesis as the take-off leg results in more efficient energy conversion for athletes with BKA compared to non-amputees during the take-off step. Due to this improved energy conversion efficiency, athletes with BKA lost less horizontal kinetic energy and consequently lost less horizontal velocity during the take-off step. Our results indicate that greater amounts of passive energy storage within the leg might be beneficial for tasks involving high collision energies like the long jump take-off step as opposed to dynamic tasks involving relatively smaller collisions, like sprinting. In fact, lower collision angles were measured during sprinting (~11°) versus jumping (~17°), in athletes with and without BKA (Fig. 4). Sprinting performance may be more closely related to generation of high leg stiffness^16,17^, high stance average vertical support forces^14,15,19^ and horizontal forces and power^13,28^. Nonetheless, the amount of passive elastic energy storage was not an independently controlled variable in this study. Therefore, the observed differences may be also related to different motor behaviours, inertial properties, or other factors.

Quantifying the potential performance advantage resulting from the use of an RSP during the take-off step and the potential performance disadvantage resulting from the use of an RSP during maximum speed sprinting is difficult and deals with a substantial amount of uncertainty. A performance advantage might result from reduced horizontal velocity losses when using an RSP for the take-off step compared to non-amputees. Whereas a performance disadvantage might result from differences in sprint mechanics forced by the use of RSPs and corresponding slower run-up and maximum sprinting speeds.

The best athlete with BKA in this study left the ground after take-off with essentially the same CoM conditions (vertical take-off velocity, horizontal and vertical take-off position) as the mean of the non-amputee sample (Table 2). Therefore, differences in the jump distances achieved between the best athlete with BKA and the average non-amputee athlete were almost entirely the result of a different horizontal take-off velocity. In this case, a performance advantage results from a lower horizontal velocity loss during the take-off step of athletes with BKA compared to non-amputee athletes. Given the initial take-off CoM conditions provided in Table 2, one can calculate the resulting flight time between take-off and landing to be 0.884 s, based on the laws of ballistic flight. The theoretical advantage (Advantage_take-off_) provided by the use of the RSP could be calculated as:

2 $${{\rm{Advantage}}}_{{\rm{take}} \mbox{-} {\rm{off}}}=0.884\,{\rm{s}}\,\cdot \,{{\rm{\Delta }}{\rm{V}}}_{\mathrm{loss}\_\mathrm{hor}}$$

Here, ∆V_loss_hor_ refers to the difference in horizontal velocity loss between an athlete with BKA and a non-amputee reference value. When taking the mean value of the non-amputee athletes from our study as the reference, ∆V_loss_hor_ equals 0.55 m/s (95% confidence interval: [0.38, 0.72], Table 2), which results in a theoretical advantage of 0.49 m (95% confidence interval: [0.34, 0.64]) during the take-off step for the best athlete with BKA.

**Figure 7:**
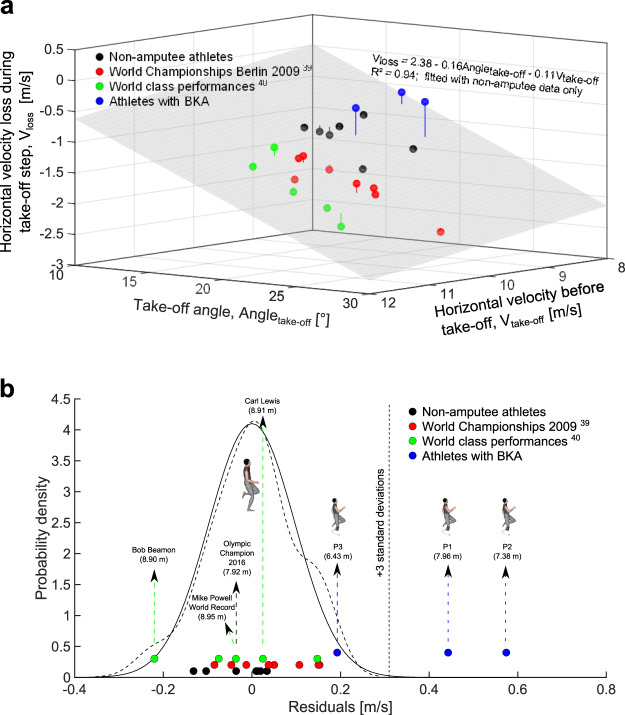
Visualization of the multiple regression analysis performed in order to provide a more general framework for the determination of the potential advantage due to the use of RSPs during the take-off step. (**a**) 3D representation of the performed multiple regression analysis using only non-amputee data as input. Different colours represent individual performance from different athletes/sources in the literature^39,40^. Vertical lines indicate residuals. The frequency distribution of these residuals is shown in (**b**). A normal distribution using the mean and standard deviation of the non-amputee residuals is plotted, next to a kernel density function calculated using the same input. The similarity between these two frequency distributions shows how well the residual data follows a normal distribution. Vertical arrows indicate individual performances of selected athletes, providing the jump distances achieved in brackets.

A problem with this approach is that it compares the data from the best athlete with BKA to a reference data set of limited size (n = 7) and that it is only valid if the take-off conditions between the athlete under consideration and the non-amputee reference data are very similar, like for the best athlete with BKA (Table 2). Nonetheless, it is well accepted that horizontal velocity losses during the take-off also increase as a function of run-up speed and take-off angles in non-amputee athletes^1,38^. In an attempt to develop a more general method to estimate a potential take-off advantage due to the use of RSPs for athletes using different approach speeds and take-off angles, we fitted a linear multiple regression model using horizontal velocity at touchdown and take-off angle as input variables to predict the horizontal velocity loss during the take-off step (R² = 0.94, Fig. 7). In this analysis, we used our non-amputee data, and data from world class athletes^39^ and from some of the best long jump performances in history from the literature^40^ (Fig. 7). Subsequently, we calculated the residuals of non-amputee athletes and athletes with BKA from the non-amputee regression plane (Fig. 7). These residuals represent the difference in horizontal velocity loss of an individual athlete compared to an average world class long jump take-off. The best athlete with BKA displayed a residual more than 4 standard deviations away from the average residual of world class non-amputee performances, which highlights that the take-off technique utilized due to low horizontal velocity loss provides an artificial performance advantage compared to non-amputee jumpers. From basic probabilistic theory, 99.7% of the data following a normal distribution are found within a range of the mean ± three standard deviations. Therefore, it seems plausible to use these values as very conservative boundaries for naturally occurring horizontal velocity losses in non-amputee athletes. The difference of the residual of the best athlete with BKA and the upper limit of these boundaries is 0.15 m/s (Fig. 7B). Implementing this value for ∆V_loss_hor_ into equation (2), a performance advantage of at least 0.13 m is a conservative estimate of the advantage during the take-off step compared to non-amputee long jumpers.

Nonetheless, this estimation is only valid for the run-up speed used by the best athlete with BKA in the present study. There is no scientific evidence that athletes with BKA follow the same relationship between approach speed, take-off angle and horizontal velocity loss as determined by us (Fig. 7) or others^2^. Therefore, future studies need to investigate in detail the relationship between horizontal velocities prior to take-off, take-off angles and horizontal velocity losses during the take-off step in athletes with BKA taking off from an RSP.

Calculating a potential performance disadvantage due to the use of RSPs is more difficult in sprinting than for the take-off step. In this study, the best athlete with and without BKA achieved very similar long jump performances (7.96 m vs. 7.92 m, respectively) with differences of 0.54 m/s in horizontal velocity immediately before the take-off step and 0.66 m/s in maximum speed sprinting. The critical question is how much of this difference was due to an intrinsically lower sprinting ability of the athlete with BKA and how much was caused by a potential disadvantage induced by the use of an RSP?

The best athlete with BKA was able to compensate for his impaired ASF application ability on his affected leg with higher ASF application on his unaffected leg; thereby, when considering both legs, overall ASF application was not reduced compared to non-amputee athletes. Nonetheless, it is conceivable that adjustments in his sprinting mechanics were necessary to realize this compensation, which might have led to negative effects on his sprinting speed. On the other hand, these adjustments might also be necessary in non-amputee athletes with ASF application asymmetry. The 9% fluctuating asymmetry found for the best athlete with BKA is inside the range of the mean ± one standard deviation (6.1% ± 3.8%) for non-amputee long jumpers reported in the present study and within two standard deviations (4.1% ± 5.2%) of the mean of a study on non-amputee treadmill sprinting^18^. This indicates that the difference in ASF application asymmetry of the best athlete with BKA is less pronounced and may be within normal ranges for non-amputees. Future studies are needed to examine the contribution of ASF asymmetry on maximum sprinting speed.

Apart from ASF asymmetry, other factors can affect sprinting performance. A study on athletes with BKA found that the dynamics of their affected legs display less dynamic stability compared to their unaffected legs and to the biological legs of non-amputee runners^41^. Furthermore, except for the first steps of a sprint, little is known about the horizontal force and power abilities and the influence of RSPs during the acceleration phase. Due to the importance of horizontal force and power application in order to reach maximum sprinting speed^13,28^, there is a clear need for further fundamental research in this area, before a valid estimation of maximum sprinting speed performance differences due to the use of RSPs can be determined. This should also address other details of the sprinting technique related to the morphological differences between legs, which might affect running performance^30–32^.

To conclude, we provide an integrated kinematic and kinetic framework for evaluating differences in performance between Olympic and Paralympic long jumpers; and as such, our results may prove useful for researchers, regulators and decision-makers in this field. We found that athletes with BKA use a slower approach speed and have slower maximum sprinting speeds compared to non-amputee long jumpers. Because of the different factors that affect maximum speed, it is not possible to estimate a potential performance disadvantage due to the use of RSPs in approach speed and maximum sprinting. Nonetheless, when taking both legs into account, the sprint mechanics of athletes with BKA were more similar to their non-amputee counterparts compared to their mechanics during the take-off step. During the take-off step, we found a different and mechanically more effective motor solution in athletes with BKA. We conservatively estimate a minimum take-off step performance advantage of 0.13 m for the best athlete with a BKA compared to non-amputees. Taking-off from an RSP allows the storage and return of a large amount of energy within the prosthesis. The energy storage and return capacities of RSPs used by athletes with BKA may exceed the biological energy storage and return capacities within the take-off legs of non-amputees, making this technique unachievable for non-amputee long jumpers. Based on our findings, future technical regulations regarding inclusion of Paralympic athletes in the Olympics should consider both the biomechanics of the final movement and the potential for preceding trade-offs, as they are identified by future research. In addition, rules committees need to take into account the comparability of biologically and technologically generated motor control solutions. Our results show that the motor solution strategy adopted by athletes with BKA during the take-off step is more effective and different from that of non-amputee athletes. Therefore, our results suggest that due to different movement strategies, athletes with and without BKA should likely compete in separate categories for the long jump.

## Methods

Data acquisition took place at the German Sport University Cologne (GSU) and the Japanese Institute of Sport Sciences (JISS). Ethical approval was obtained from the ethical committee of the German Sport University (approval number: 040/2016). The protocol was performed in accordance with the relevant ethical guidelines and regulations, based on the Declaration of Helsinki.

### Subjects

Two groups of subjects were included. Group 1 comprised three of the world’s best long jumpers with a unilateral below the knee amputation (BKA; age: 26 ± 1.7 years; body mass (including socket and prosthesis): 78.7 ± 9.76 kg; standing height: 1.83 ± 0.04 m; long-jump personal record [PR]: 7.43 ± 0.99 m), and included the world-record holder (International Paralympic Committee T44 classification, unilateral BKA). Group 2 comprised seven non-amputee long jumpers (age: 24.6 ± 2.5 years; body mass: 80.1 ± 6.22 kg; standing height: 1.82 ± 0.07 m; PR: 7.65 ± 0.65 m), who were competitive at international, national and regional levels. All subjects participated voluntarily and gave written informed consent. Consent to publish subject photos was obtained from each subject.

### Treatments

Prior to data collection, the anthropometrics of each athlete were recorded according to the reference handbook of the ALASKA modelling system^42^. Furthermore, detailed measurements of different portions of the prosthesis of each subject with a BKA were taken. These included weight and geometry measurements (lengths, circumferences, curvatures, etc.). After an individual, competition-specific warm-up, all athletes performed long jumps and maximum speed sprints aimed at achieving their best performance. Athletes were asked to use their individual competition-specific approach run for the long jump, and to use an approach run that enabled them to reach a constant maximum velocity in the measuring volume for the maximum speed sprints. Two athletes completed both movement trials on the same day, but all others performed the jumping and sprinting trials on different days to avoid any potential effects of fatigue.

### Kinematics

Retro-reflective spherical markers (10 mm; Twist, ILUMARK GmbH, Feldkirchen, Germany) were fixed to anatomical landmarks on the athlete’s body and prosthesis using ph-neutral double-sided tape. In total, 55 (non-amputee) or 83 (BKA) markers were used (Figure S5).

Marker trajectories for the long jump and sprinting were captured by means of an infrared camera system (250 Hz; Vicon, Oxford, UK; long jump: 20 cameras (MX40); sprinting: 14 cameras (T-Series)). One static trial in upright standing position was captured and used to define the neutral position for all joints. Athletes with BKA placed their unaffected leg on a wooden block during the static trial so that there were no differences in hip height between legs and to ensure comparability with the non-amputees. Approach-run and sprinting velocities were recorded using a laser gun (100 Hz, LAVEG, Jenoptik, Jena, Germany). Step frequency and flight time during sprinting were recorded using an optical measurement system (1000 Hz, OPTOJUMP, Microgate, Bolzano, Italy) with a length of 13 m. Additionally, three high speed video cameras (100 Hz; Basler, Ahrensburg, Germany) were used to take qualitative videos of the take-off phase of the jump in order to ensure that each athlete landed on the force plate(s).

For two subjects (one non-amputee and one with BKA), motion-capture data was only obtained from the jumping trial, but not from the maximum sprinting trial. Therefore, in sprinting trials, only the running speed and ground reaction force (GRF) measurements are included in the analysis of these athletes.

### Kinetics

GRFs were captured simultaneously with kinematic data using piezo-based force plates (1000 Hz; Kistler Instrumente AG, Winterthur, Switzerland). For the long jump, GRFs were captured during the take-off step. Therefore, a force plate (40 × 60 cm) covered with a wooden take-off board (GSU) or a force plate (90 × 60 cm) covered with the same tartan surface as the run-up (JISS) were used. For the sprinting trials, four (GSU) or six (JISS) force plates (90 × 60 cm) were used that were mounted flush with the floor and covered with the same tartan surface as the run-up.

### Post-processing

All motion capture data were visually checked for valid force plate contacts using the high-speed video. Three dimensional marker coordinates were reconstructed and labelled within the same software (Nexus 2.3, Vicon Motion Systems, Oxford, UK). Small gaps (<10 frames) within the marker trajectories were filled using implemented algorithms. Marker coordinates and GRFs were both filtered using the same filter (4^th^ order recursive digital Butterworth filter; 50 Hz cut-off frequency) in order to avoid artefacts within the model-based inverse dynamics calculations^43,44^.

### Ground reaction forces

Bodyweight normalized stance average vertical support force (ASF) was calculated by taking the average of the filtered vertical GRF over the entire stance phase. Bodyweight was measured from the static standing reference measurement. Stance phases were defined using a 20 N threshold of the resultant GRF. Vertical and net horizontal impulses were determined for each stance period by numerical integration of vertical and horizontal GRF curves with respect to time. Impulses were also normalized by bodyweight (BW).

### Model calculations

Inverse dynamics calculations were executed using a modified version of the full-body model, Dynamicus (ALASKA, Advanced Lagrangian Solver in Kinetic Analysis, Institute of Mechatronics, Chemnitz, Germany^42^). The prosthesis was modelled as two rigid bodies with a ball joint connection. The prosthetic joint was defined by two markers placed at the medial and lateral edge of the prosthesis and positioned with respect to the most posterior point of the prosthesis, which coincided with the point of highest curvature of the prosthesis^45^. While this approach has been utilized in previous publications^45,46^, other approaches for modelling the total power of prosthetic below-knee structures exist. For example, a unified deformable segment model has been used for quantifying the total power of the entire prosthesis^47^. It is possible that some degree of error in below knee energy computations resulted from the chosen method^47^. All body markers were rigidly attached to the corresponding segments of the full-body model using their 3D coordinates, which were obtained from the standing reference measurement.

The motion of the model was calculated using a standard inverse kinematics procedure. Within these calculations, the (measured) tracking markers were mathematically optimized using a weighted square deviation of the position of the body mounted (model) markers from their corresponding tracking markers. The anthropometric segmental parameters were taken from previously established equations^48–50^.

The segmental coordinate systems were defined using the standing reference measurement and were attached to each segment. The hip joint centres (HJC) were estimated using a regression equation provided by the software. The knee (KJC) and ankle joint centres (AJC) were defined as the central points between the medial and lateral femoral condyles and the medial and lateral malleoli markers, respectively. The joint centre of the metatarsophalangeal joints (MJC) were defined as the midpoint between the 5^th^ and 1^st^ metatarsal head markers. The external joint moments were calculated using the inverse dynamics method and are described in the distal segmental coordinate system.

The inertia parameters of the prosthesis were calculated by dividing it into 9 cuboids. The geometric dimension of each cuboid was obtained by measuring the width, length and thickness of the corresponding region of the prosthesis. The geometrical model of the prosthesis was designed in a way that prosthetic joints were positioned between two cuboid segments of the prosthesis. The volume of the whole prosthesis was calculated by summing the volumes of individual cuboid segments. The density of the prosthesis was assumed to be homogenous and was estimated by dividing the mass of the prosthesis by its volume. Consequently, each cuboid was assigned a definite mass. The moment of inertia of each cuboid segment was estimated using standard equations^51^.

The centre of mass (CoM) of the whole modified full-body model was estimated via the anthropometric dimensions of the biological parts of the model combined with the properties and dimensions of the prosthesis. Joint power was calculated using the following equation^23^:

3 $${P}_{j}={M}_{j}\times {\omega }_{j}$$

P is the power of joint j, M_j_ represents the resultant internal moment of joint j and ω_j_ represents the angular velocity of the joint j. Negative joint power was defined as energy absorption, while positive joint power was defined as energy generation. Joint work was calculated by numerically integrating joint power over time. Joint work was the sum of the work of all three planes of motion.

CoM velocity in sprint trials was calculated by numerical differentiation of the raw position data obtained from the laser gun system. CoM velocity was smoothed using a recursive digital Butterworth low-pass filter (4^th^ order, 1 Hz cut-off). From the filtered CoM velocity, peak velocities were obtained during the sprinting and jumping trials.

Potential CoM energy (E_pot_) was calculated as follows:

4 $${E}_{pot}=mgh$$

m is the body mass, g is gravitational acceleration and h is the CoM height with respect to the global laboratory reference frame, originating at the surface of the running track. Horizontal and vertical kinetic CoM energy (E_kin_hor_, E_kin_vert_) were calculated according to the following equations, respectively:

5 $${E}_{kin\_hor}=\frac{m{v}_{hor}^{2}}{2}$$

6 $${E}_{kin\_vert}=\frac{m{v}_{vert}^{2}}{2}$$

v_hor_ and v_vert_ represent the horizontal and vertical CoM velocities, respectively. Total CoM energy was calculated as the sum of the potential CoM energy and the two components of kinetic CoM energy.

Averaged weighted collision angles, determined by the angle between the GRF and CoM velocity vectors, were calculated for the energy absorption phases during maximum sprinting and for the jump take-off using the formulae provided in reference^22^.

For both athletes with and without an amputation, three joints contributing to the energy exchange of the system were defined: hip, knee and ‘below knee’. For athletes with BKA, the below knee joint represents the energy exchange of the prosthesis, whereas for the non-amputees, the below knee joint combines the energy exchange of the ankle and metatarsophalangeal joints. Joint energy was calculated by numerically integrating the power-time curve and summing all three planes of motion. However, in accordance with common practice^23^, the energy of the metatarsophalangeal joint only represents the energy exchange within the sagittal plane for time periods in which the point of GRF application is anterior to the metatarsophalangeal-joint centre.

### Spatiotemporal parameters

Step frequency (Freq_step_) was calculated as

7 $$Fre{q}_{step}=2\cdot Fre{q}_{stride}$$

Stride frequency (Freq_stride_) was calculated as:

8 $$Fre{q}_{stride}=\frac{1}{{t}_{stride}}$$

Stride time (t_stride_) was defined as the time interval from touchdown of one foot on the ground to the next ipsilateral touchdown. This time interval included a left and right contact phase and two aerial phases, from the left leg to the right leg and vice versa.

### Jump distance calculations

For all jump trials, we assumed a parabolic flight curve of the CoM after take-off; that drag forces due to air resistance were negligible, and we did not consider landing technique.

9 $$Co{M}_{flight\_AP}(t)=Co{M}_{0\_AP}+{v}_{com0\_AP}\cdot t$$

10 $$Co{M}_{flight\_Vert}(t)=Co{M}_{0\_Vert}+\frac{g{t}^{2}}{2}+{v}_{com0\_Vert}\cdot t$$

The starting point of the flight path was defined by the vertical (CoM_0_vert_) and horizontal (CoM_0_AP_) CoM positions at final contact with the ground, while the resulting CoM velocity (v_com0_res_) and take-off angle (α) were calculated from the initial flight phase vector of the CoM, which was determined by subtracting the initial CoM position from the CoM position 25 ms after the last contact with the ground. Jump distance was defined as the distance from the most anterior point of the foot or prosthesis during take-off ground contact to the intersection between the CoM flight path and the ground^52^. We used this approach to avoid taking into account the effects of landing technique on long jump performance. There was a strong correlation between the jump distances determined as described above and the jump distances measured with a tape measure (r = 0.99, p < 0.001; average difference: −0.36 m).

### Take-off performance advantage calculations

The analysis of a potential performance advantage was focussed on the horizontal velocity loss of the CoM during the take-off step. More specifically, we assessed the differences between the horizontal velocity losses of the best athlete with BKA compared to reference data of non-amputee athletes. Horizontal velocity losses during the take-off step are strongly related to run-up speed and take-off angle in non-amputee athletes^1,38^. Therefore, we fitted a multiple regression model using horizontal velocity immediately prior to the take-off step and take-off angle as input variables to predict the horizontal velocity loss during the take-off step (Fig. 7). The model shared 94% of the variance with the measured values of horizontal velocity losses. Only data from non-amputee athletes were used for the statistical modelling. In order to improve the inferential conclusions drawn from the model, we added previously published data from the literature using 16 mm high-speed cine cameras and high-speed video cameras to calculate CoM velocity and take-off parameters^39,40^. The data from the literature represent some of the best long jump performances in the history of the sport obtained during World Championships and Olympic competitions. In general, the approach speeds are faster compared to data from the present study, which were captured in a laboratory setting. Nonetheless, the corresponding horizontal velocity losses during take-off are higher indicating that these data sets, collected when athletes were at their peak athletic ability and peak motivation, have the same underlying relationship between run-up speed, take-off angle and horizontal velocity loss. This is also indicated by the high R² value obtained for the multiple regression model.

In the second step of the analysis we fitted a normal distribution to the residuals of the multiple regression model. To confirm the correspondence of the residual data with this normal distribution we used a kernel fitting technique (Fig. 7b). The resultant fit qualitatively corresponded well with the fitted normal distribution (Fig. 7b). Subsequently, we used a threshold value of three standard deviations from the mean as a conservative estimate of typically occurring deviations from the predicted horizontal velocity losses in non-amputee athletes. Any value outside of this range was considered an artificially induced difference. From this, we calculated the artificially induced horizontal velocity loss reduction of the best athlete with BKA, which was subsequently used in equation (2) to calculate the performance in distance jumped.

### Statistics

Due to low sample sizes, we used a non-parametric test (Wilcoxon rank sum test) in order to compare the results of non-amputee athletes to athletes with BKA. While taking this low sample size into account, we set the level of significance to 0.10.

Furthermore, the absolute and relative differences (in % of the non-amputee value) between the best non-amputee athlete and the best athlete with BKA are described in order to gain insight into differences at the very top level of performance.

Pearson correlation analyses and a multiple linear regression analysis were performed after checking for the respective assumptions made by these tests.

### Data and code availability

All raw data and custom written code for the analysis of the data is available from https://dshs-koeln.sciebo.de/index.php/s/K8wY40iRvJyhqnH. All code was created using Matlab (R2015b, The Mathworks, Natick, MA, USA).

## Electronic supplementary material

Supplemetary Information
