# Supplementary material for: Elite long jumpers with below the knee prostheses approach the board slower, but take-off more effectively than non-amputee athletes
Source: Sci Rep. 2017 Nov 22;7:16058. doi: 10.1038/s41598-017-16383-5 (PMC5700183; doi:10.1038/s41598-017-16383-5)
Supplement: Supplementary file 1 — Supplemetary Information [file 41598_2017_16383_MOESM1_ESM.pdf]

Supplementary Information

Elite long jumpers with below the knee prostheses approach the board slower, but take-off more effectively than non-amputee athletes

Steffen Willwacher, Johannes Funken, Kai Heinrich, Ralf Müller, Hiroaki Hobara, Alena M. Grabowski, Gert-Peter Brüggemann, Wolfgang Potthast

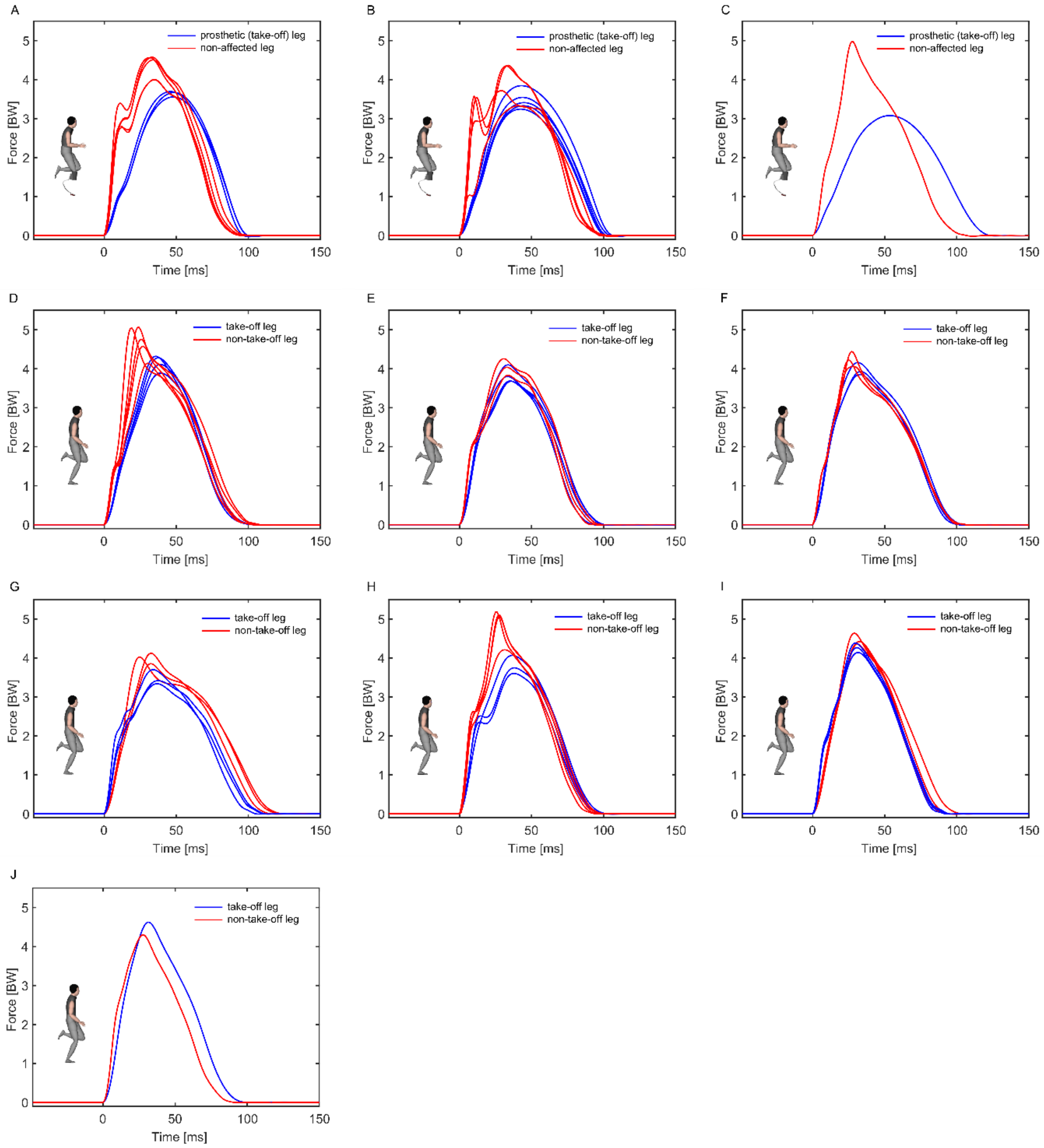

Fig. S1: Individual athlete's vertical ground-reaction force (GRF) curves during maximum-speed sprinting in units of bodyweight (BW). A-C, Athletes with below-the-knee amputation (BKA). D-J, Non-amputee athletes. The take-off leg is represented in blue and the non-take-off leg in red. In athletes with BKA, the take-off leg is also the affected leg. For biological legs in both sets of athletes, the GRF curve is skewed left towards the earlier period of the stance phase (ground contact). In contrast, the affected legs of athletes with BKA exhibit a more temporally symmetrical, near half-sinusoidal, curve that is typical of classical spring-mass behaviour.

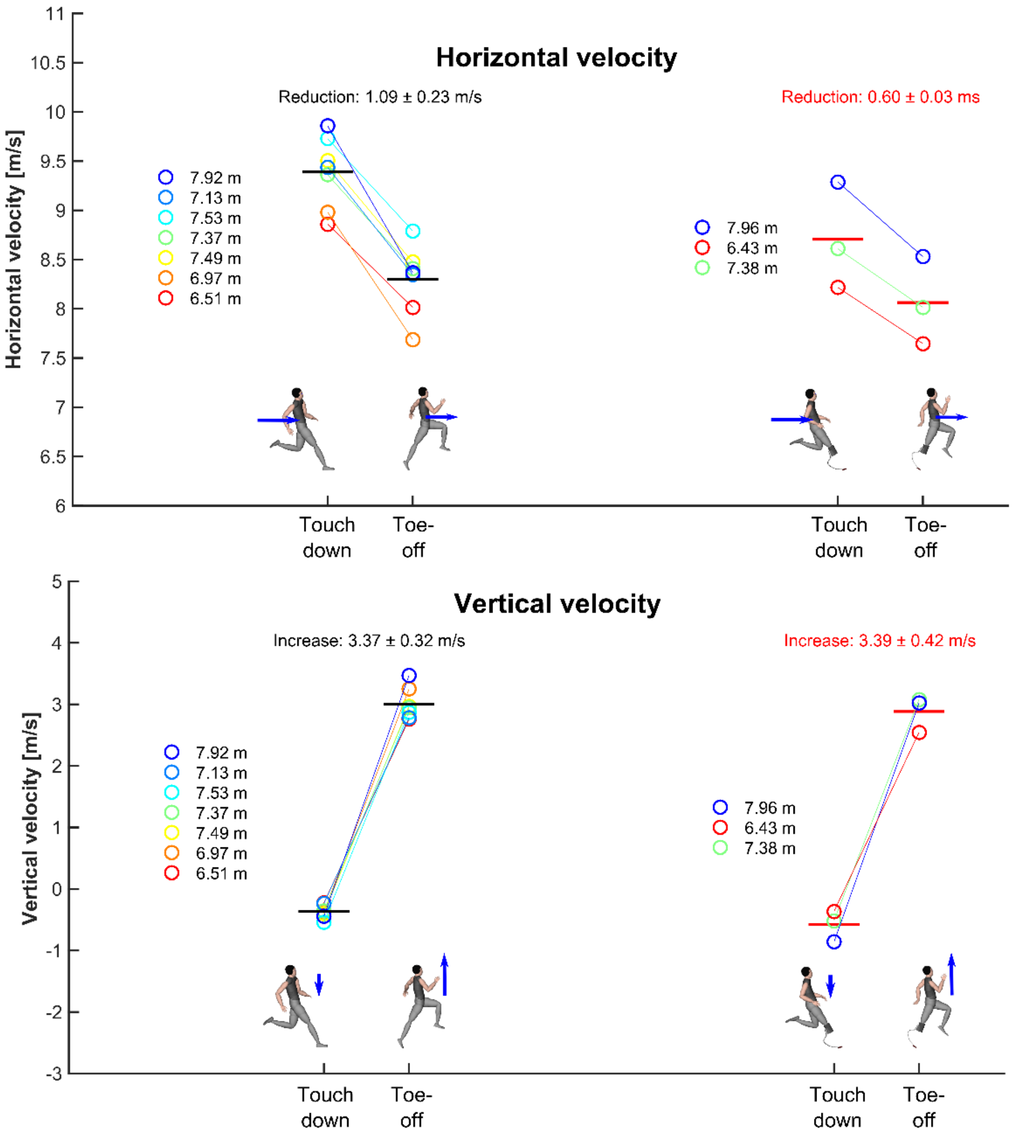

Fig. S2: Centre-of-mass velocity changes during the take-off step in the horizontal (top) and vertical (bottom) directions. Athletes with below-the-knee amputation (BKA) have a slower horizontal run-up velocity, but they lose less horizontal velocity during the take-off. No differences with respect to vertical velocity were identified. Blue arrows represent the vertical and horizontal velocity vectors during touch-down and toe-off.

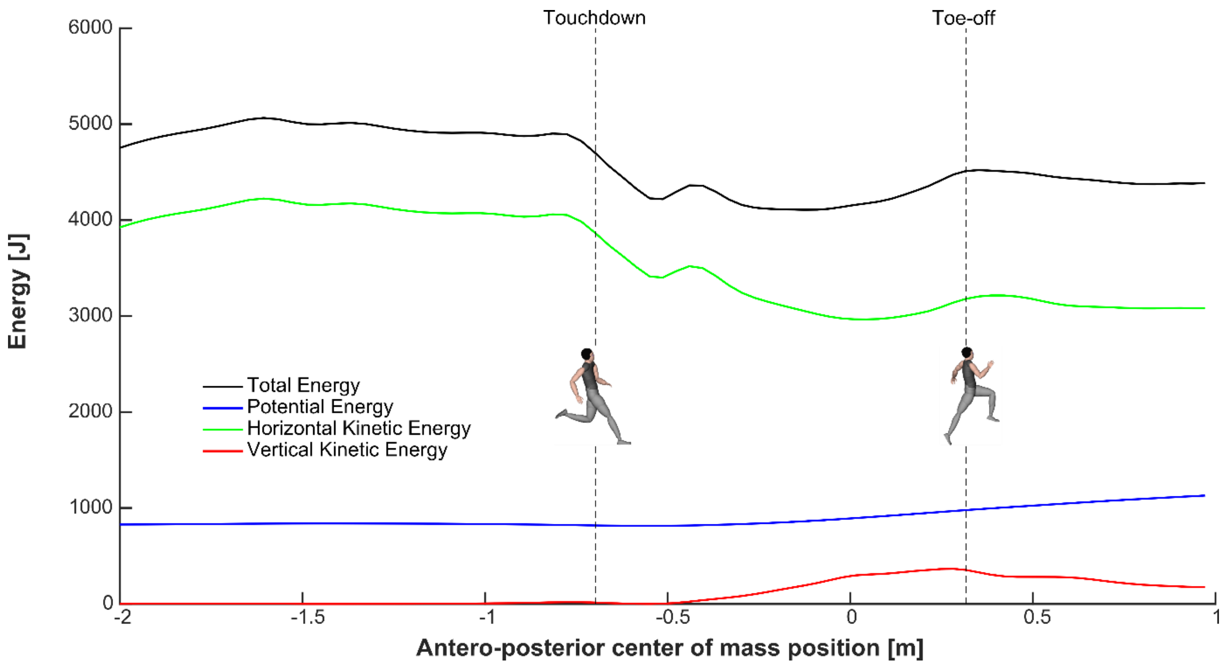

Fig. S3: Total, potential, horizontal kinetic and vertical kinetic centre of mass (CoM) energy from a representative non-amputee during the take-off step. Total CoM energy is the sum of the potential and kinetic energies within the sagittal plane of motion. Horizontal kinetic energy was the dominant component of the total CoM energy. While horizontal CoM energy decreased during the take-off step (from touchdown to toe-off), potential and vertical kinetic energy increased until the end of the take-off.

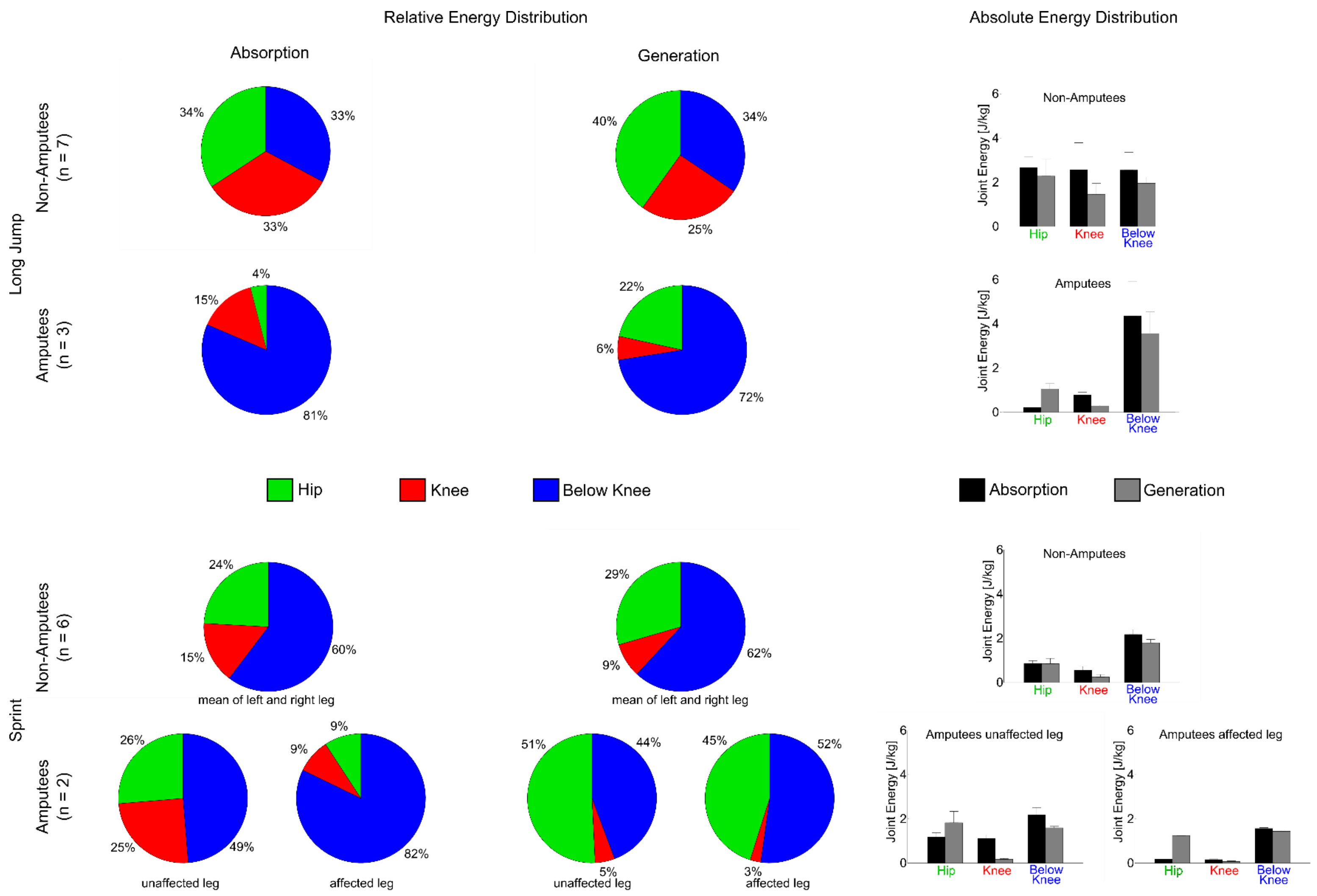

Fig. S4: Pie charts showing the energy distribution of the hip, knee, and 'below knee' (ankle and the metatarsophalangeal) joints relative to the total amount of joint energy generated (left) and absorbed (right). Bar graphs show the total energy distribution of the joints normalized to body mass. Top rows show energy distribution during the take-off step of the long jump. Bottom rows show the energy distribution during the stance (ground contact) phase of sprinting.

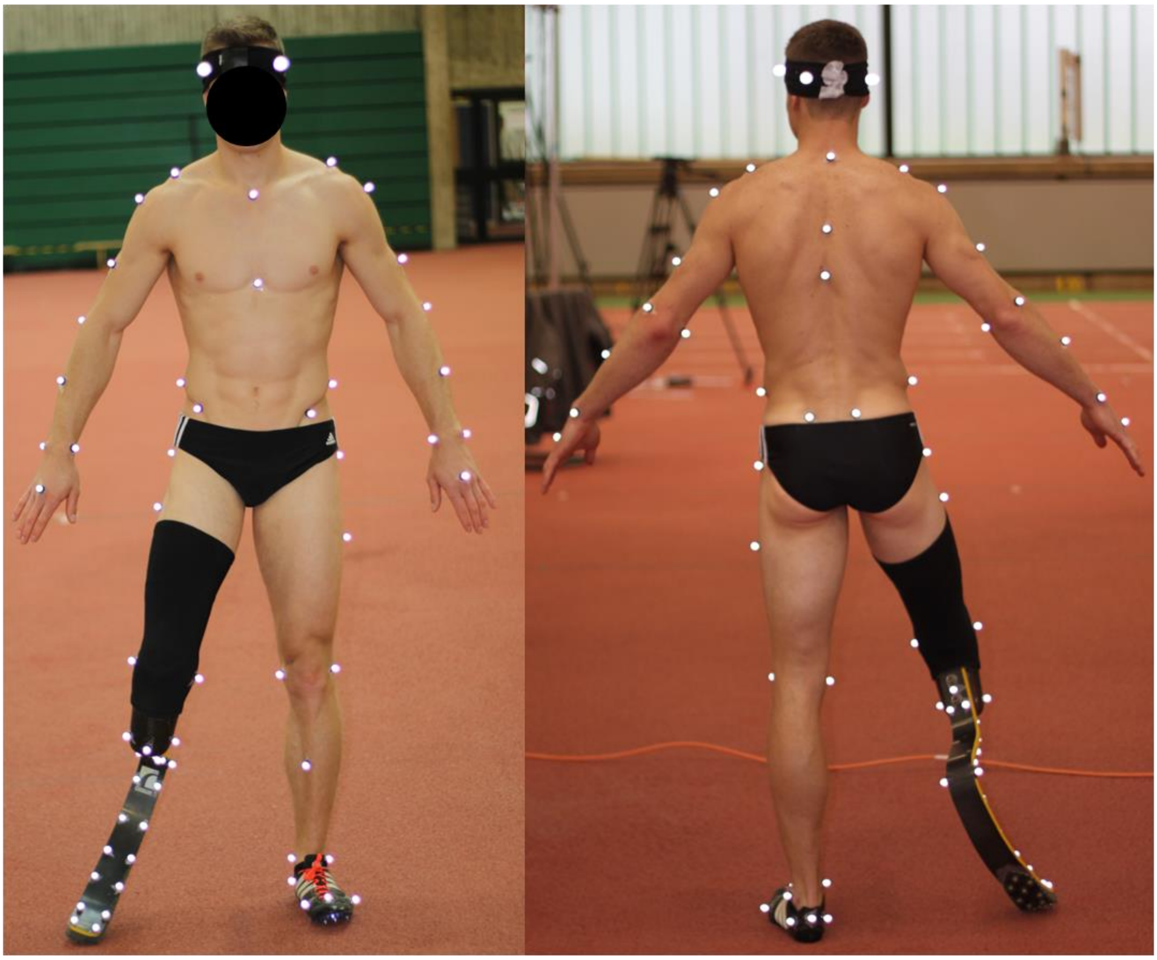

Fig. S5: Marker set used in the study. The marker set of the athletes with BKA was the same as for non-amputees, except for in the area below the knee of the affected leg. Marker arrangement was bilaterally symmetrical across the legs of non-amputees.
